# Implementing a toolkit for the prevention, management and control of carbapenemase-producing Enterobacteriaceae in English acute hospitals trusts: a qualitative evaluation

**DOI:** 10.1186/s12913-019-4492-4

**Published:** 2019-10-12

**Authors:** Annegret Schneider, Caroline Coope, Susan Michie, Richard Puleston, Susan Hopkins, Isabel Oliver

**Affiliations:** 1 University College London, Gower St, Bloomsbury, London, WC1E 6BT UK; 2 National Institute for Health Research Health Protection Unit in Evaluation of Interventions, Bristol Medical School, University of Bristol, Bristol, BS8 2PS UK; 3 Field Service South West, National Infection Service, Public Health England, 2 Rivergate, Bristol, BS1 6EH UK; 4 Field Service East Midlands, National Infection Service, Public Health England, Nottingham, NG24LA UK; 5 Division of Healthcare-Associated Infection and Antimicrobial Resistance, National Infection Service, Public Health England, London, UK

**Keywords:** Antimicrobial resistance, Infection prevention and control, Carbapenem, Hospital infection, Implementation science, Theory-driven evaluation, Complex interventions, Guideline, Acute care settings

## Abstract

**Background:**

Antimicrobial resistance is an increasing problem in hospitals world-wide. Following other countries, English hospitals experienced outbreaks of carbapenemase-producing Enterobacteriaceae (CPE), a bacterial infection commonly resistant to last resort antibiotics. One way to improve CPE prevention, management and control is the production of guidelines, such as the CPE toolkit published by Public Health England in December 2013. The aim of this research was to investigate the implementation of the CPE toolkit and to identify barriers and facilitators to inform future policies.

**Methods:**

Acute hospital trusts (*N* = 12) were purposively sampled based on their self-assessed CPE colonisation rates and time point of introducing local CPE action plans. Following maximum variation sampling, 44 interviews with hospital staff were conducted between April and August 2017 using a semi-structured topic guide based on the Capability, Opportunity, Motivation and Behaviour Model and the Theoretical Domains Framework, covering areas of influences on behaviour. Interviews were audio-recorded, transcribed verbatim and analysed using thematic analysis.

**Results:**

The national CPE toolkit was widely disseminated within infection prevention and control teams (IPCT), but awareness was rare among other hospital staff. Local plans, developed by IPCTs referring to the CPE toolkit while considering local circumstances, were in place in all hospitals. Implementation barriers included: shortage of isolation facilities for CPE patients, time pressures, and competing demands. Facilitators were within hospital and across-hospital collaborations and knowledge sharing, availability of dedicated IPCTs, leadership support and prioritisation of CPE as an important concern. Participants using the CPE toolkit had mixed views, appreciating its readability and clarity about patient management, but voicing concerns about the lack of transparency on the level of evidence and the practicality of implementation. They recommended regular updates, additional clarifications, tailored information and implementation guidance.

**Conclusions:**

There were problems with the awareness and implementation of the CPE toolkit and frontline staff saw room for improvement, identifying implementation barriers and facilitators. An updated CPE toolkit version should provide comprehensive and instructive guidance on evidence-based CPE prevention, management and control procedures and their implementation in a modular format with sections tailored to hospitals’ CPE status and to different staff groups.

**Electronic supplementary material:**

The online version of this article (10.1186/s12913-019-4492-4) contains supplementary material, which is available to authorized users.

## Background

Carbapenemase-producing Enterobacteriaceae (CPE), Gram-negative bacteria with resistance to carbapenem antibiotics, are an increasing problem world-wide [1–3]. Carbapenems are powerful broad spectrum antibiotics and one of the last effective treatments for multi-resistant bacteria [4]. In some European countries, CPE are endemic, particularly in southern countries such as Greece and Italy [5, 6]. Evidence suggests that hospitalisation, especially when occurring in countries with high numbers of patients colonised or infected with CPE, is a key risk factor for CPE spread and associated deaths [7–9]. The United Kingdom has seen a continual increase in incidence of detected colonisations and infections since 2008 with fewer than five confirmed cases between 2004 to 2007 followed by 23 in 2008, 73 in 2009, 333 in 2010, and 561 in 2011 [5].

In December 2013, as part of the response to a number of CPE outbreaks in acute hospitals in England, Public Health England (PHE) published a guidance document, the “Acute trust toolkit for the early detection, management and control of carbapenemase-producing Enterobacteriaceae” and hereafter referred to as the CPE toolkit [10]. Acute trusts are organisational structures of the English National Health Service (NHS), which manage one or more acute care hospitals (secondary and tertiary) with a single management board. The CPE toolkit was produced to offer guidance on CPE, transmission risk factors, early recognition and prevention of transmission to inform the development of trust-specific plans. A literature search was conducted to identify and review relevant publications, documents and guidelines focusing on lessons learned from countries that had experienced CPE outbreaks previously. A question and answer section of the toolkit was based on real questions asked by health care professionals as well as further anticipated questions. The CPE toolkit was formally launched in March 2014 with an NHS England Patient Safety Alert requiring acute trusts to have a local CPE plan in place by June 2014 [11].

The aim of this implementation evaluation was to investigate the awareness of the CPE toolkit amongst frontline staff with specific infection prevention and control responsibilities (IPC), such as IPC nurses or microbiologists, as well as non-IPC specialist frontline staff, including nurses and doctors. Further, the implementation of procedures recommended by the CPE toolkit and the processes by which the CPE toolkit may have influenced clinical practice were examined [12, 13]. Implementation evaluations are particularly important for interventions like the CPE toolkit, which facilitate adaptation to the local context [14, 15]. By encouraging the development of local plans implemented process may vary between trusts, for instance use of risk-based screening of all patients versus limited screening for specific wards. Examining contextual influences, differences between the recommended and implemented processes and identifying key implementation facilitators and barriers from the perspective of frontline staff should help improve and strengthen future versions of the CPE toolkit and inform other policies.

## Methods

### Implementation evaluation frameworks

The Behaviour Change Wheel (BCW), a comprehensive framework for developing and evaluating behaviour change interventions, was used to theoretically inform the evaluation [16]. It describes seven broad policy level options through which behaviour can be influenced: fiscal measures, regulations, service provision, legislation, communication and marketing, environmental and social planning, and guidelines. To target capabilities, opportunities and motivation to perform behaviours (COM-B model), policy interventions support specific intervention functions, for example education, persuasion, incentivisation, coercion, training, restriction and environmental restructuring. Mapping onto the COM-B model, the Theoretical Domains Framework (TDF) breaks down the capability, opportunity and motivation domains further, outlining 14 areas of influence on professional behaviour, for example improving skills, changing beliefs about consequences or social influences [17, 18]. We examined which influences were relevant to the CPE toolkit implementation and what behaviour change pathways applied according to frontline staff. The evaluation built on empirical findings from a 2016 online survey of executive acute trust staff on organisational level perceptions of the CPE toolkit implementation, which suggested awareness of the CPE toolkit, but a lack of confidence in its practical value [19]. Additional file 1 illustrates how the evaluation frameworks informed the following research process.

### Design

A qualitative design was used to gain a contextual understanding of CPE awareness amongst frontline staff and their experiences of the CPE toolkit implementation [14, 20, 21]. The evaluation protocol was reviewed by the University of Bristol Research Ethics Committee, classifying this health service evaluation as an audit of existing NHS procedures [22].

### Setting

The evaluation was conducted in acute hospital trusts in England, the setting for which the CPE toolkit was developed. At the time of this research, there were 154 English acute trusts. Trusts that had given their consent to be approached for further evaluation activities (59 of 99 respondents) in the prior online survey [19] were purposively sampled to ensure a diverse sample [23–25]. The sampling frame focused on capturing all potential combinations of two main sampling criteria, CPE colonisation rates and time point of introducing local CPE plans, and on including large, medium and small trusts from each commissioning region. All trusts had local CPE plans in place, but the time point of initial introduction varied from prior to the CPE toolkit publication, as early as 2014, to late adoptions (2015/16). CPE colonisation, a precursor for infection [26], correlated with CPE infection rates (χ^2^ = 116.49; *p* < 0.001) and ranged from no cases, to some (1–10), and many CPE colonisation rates (> = 11) [19].

A personal access strategy [27, 28] was employed, gaining access through gatekeepers such as the local infection prevention and control teams (IPCT) and antimicrobial resistance (AMR) leads or through executive members of staff that participated in the survey. Following a maximum variation sampling approach to explore different views and experiences and to maximise the chance of discovering differences between staff with a range of roles and responsibilities [29–31], gatekeepers identified interview participants from two staff groups targeted by the CPE toolkit: 1) staff with specialist IPC responsibilities (e.g. IPC directors, lead IPC nurses or consultant microbiologists) as key informants [32], hereafter called IPC frontline staff, and 2) non-IPC frontline staff (e.g. staff nurses, ward managers and doctors) caring for patients, hereafter called general frontline staff. Settings included low CPE risk but high patient throughput wards, such as admission and acute medicine, as well as specialised, high CPE risk wards, like intensive care and surgical wards. Depending on trust size and speciality, the number of staff available for interviews varied, ranging from two to five participants per trust (Additional file 2) with at least one person from each group (Table 1). Out of the 14 trusts approached, 12 agreed to participate and 44 interviews were conducted (Table 2). Inability to spare staff time was given as reason for non-participation.

**Table 1:** Interviewed staff characteristics

Staff characteristics	Total (*N* = 44)	Face-to-Face (*N* = 25)	Telephone (*N* = 19)
Gender:			
Female	31 (70%)	18 (72%)	13 (68%)
Male	11 (25%)	6 (24%)	5 (26%)
Rather not say	2 (5%)	1 (4%)	1 (5%)
Age:			
20–29 years	6 (14%)	6 (24%)	0
30–39 years	6 (14%)17 (39%)	4 (16%)	2 (11%)
40–49 years	10 (23%)	9 (36%)	8 (42%)
50–59 years	2 (5%)	5 (20%)	5 (26%)
60–69 years	3 (7%)	0	2 (11%)
I would rather not say		1 (4%)	2 (11%)
Role / level:			
IPC^a^ frontline staff	23 (52%)	12 (48%)	11 (58%)
General frontline staff	21 (48%)	13 (52%)	8 (42%)
Area of responsibility:			
Developing IPC^a^ procedures	30 (68%)	17 (68%)	13 (68%)
Developing admission procedures	21 (48%)	12 (48%)	9 (47%)
Developing discharge/transfer procedures	18 (41%)	10 (40%)	8 (42%)
Direct patient care	25 (57%)	13 (52%)	12 (63%)
Admitting patients	14 (32%)	7 (28%)	7 (37%)
Discharging patients	14 (32%)	7 (28%)	7 (37%)
Cleaning activities	20 (45%)	14 (56%)	6 (32%)
Ward/department:			
IPC^a^	16 (36%)	10 (40%)	6 (32%)
Microbiology	7 (16%)	2 (8%)	5 (26%)
Emergency unit	4 (9%)	3 (12%)	1 (5%)
Intensive Care	4 (9%)	2 (8%)	2 (11%)
Acute assessment	2 (5%)	1 (4%)	1 (5%)
Burns and plastic surgery	2 (5%)	2 (8%)	0
Gastroenterology	2 (5%)	1 (4%)	1 (5%)
Haematology	2 (5%)	0	2 (11%)
Pathology	2 (5%)	2 (8%)	0
Haemodialysis	1 (2%)	1 (4%)	0
Surgery	1 (2%)	1 (4%)	0
Transplant unit	1 (2%)	0	1 (5%)
Time at ward/department	0.4–33.25 years (median = 7; IQR = 6.25)	0.5–33.25 years (median = 6; IQR = 9.75)	0.4–20 years (median = 8.5; IQR = 8.21)
Time at trust	0.5–36 years (median = 9.92; IQR = 18.5)	0.5–33.25 years (median = 7; IQR = 16.5)	2.72–36 years (median = 12.96; IQR = 21.5)

Percentages may not total 100 due to rounding or multiple response option

^a^IPC = Infection prevention and control

**Table 2:** Participating trusts’ characteristics

Trust characteristics	Total (*N* = 12)	Face-to-Face (*N* = 6)	Telephone (*N* = 6)
CPE colonization:			
None	3 (25%)	1 (17%)	2 (33%)
Some cases (1–10)	4 (33%)	1 (17%)	3 (50%)
Many cases (> = 11)	5 (42%)	4 (67%)	1 (17%)
Local CPE management plan:			
Pre-CPE toolkit	3 (25%)	1 (17%)	2 (33%)
Early adopters (2014)	5 (42%)	3 (50%)	2 (33%)
Late adopters (2015–16)	4 (33%)	2 (33%)	2 (33%)
Region:			
London	2 (17%)	1 (17%)	1 (17%)
South of England	3 (25%)	3 (50%)	0
Midlands and East of England	3 (25%)	2 (33%)	1 (17%)
North of England	4 (33%)	0	4 (67%)
Capacity / size^a^:			
Large	2 (17%)	1 (17%)	1 (17%)
Medium	2(17%)	1 (17%)	1 (17%)
Small	2 (17%)	2 (33%)	0
Specialist / Multi-service	2 (17%)	1 (17%)	1 (17%)
Teaching	4 (33%)	1 (17%)	3 (50%)

Percentages may not total 100 due to rounding or multiple response option

^a^Small denotes an annual income ≤ £244 m, medium £245 m - £327 m and large ≥ £328 m (Source: NHS Digital)

### Interview procedures

An interview topic guide (Additional file 3) was developed by the multidisciplinary research team, piloted with participants at two acute trusts and iteratively adapted over the course of the interviews as new insights emerged [21, 33, 34]. Semi-structured interviews ensured detailed information relevant to the evaluation questions were captured while allowing participants to raise additional topics of importance [24, 25]. Participants were asked to reflect on any ‘significant changes’ [35, 36] concerning CPE prevention, management and control since 2014 when the CPE toolkit was launched nationally. Questioning not referring explicitly to the CPE toolkit initially was aimed at identifying changes in the wider context and how much importance participants ascribed to the toolkit, if they were aware of it. Aspects related to potential behaviour change pathways and context factors were explored with questions based on the TDF [18]. As visual stimulus to prompt further insight into participants’ experiences and views on recommended procedures [14, 37], participants were asked to comment on the patient management steps specified in a flowchart included in the CPE toolkit. Interviews were conducted in accordance with data protection and confidentiality regulations, by briefing participants and obtaining informed consent. As this evaluation was commissioned by the same group at PHE who commissioned the CPE toolkit, interviewers emphasised their independence of the evaluation group to counter response biases. Depending on participants’ preferences and availability, interviews were conducted between April and August 2017 either face-to-face during one-day site visits or by telephone over a maximum period of one month (Table 1). Both interviewers (AS, CC) were women with a PhD in health science (health psychology, epidemiology) with prior qualitative research experience. Interview times ranged from nine to 108 min with an average length of 26 min. The average interview length was similar for face-to-face (27 min) and telephone interviews (24 min).

### Analysis

The interviews were digitally recorded on an encrypted recording device and transcribed verbatim. The transcripts were imported into NVivo Version 12 [38] and analysed following thematic analysis steps [39] using the constant comparison technique, coding the material line-by-line [40]. To ensure reliability and validity of the analysis [21, 30, 41], the first five transcripts were independently double-coded (AS, CC). Potential discrepancies were discussed to reach consensus and a coding framework was developed using a hybrid approach [42], combining data-driven inductive codes and deductively developing categories based on the Medical Research Council process evaluation guidelines [12] and procedures outlined by the CPE toolkit [10]. The first author applied the agreed coding framework to the remaining transcripts. Themes were converted into thematic networks [43], mapping implementation barriers and facilitators according to COM-B model categories for behavioural influences [16]. Further procedures employed to ensure quality of the data analysis included discussions of findings within the multidisciplinary team, memo writing and keeping reflective and decision logs throughout the data collection and analysis phase, grounding findings in the data through anonymised participant quotes and transparent reporting in line with recognised standards [25, 44].

## Results

Table 3 summaries results concerning awareness and reach of the CPE toolkit, the implementation and impact pathway and views, responses and improvement suggestions, followed by corresponding participant quotes.

**Table 3:** CPE toolkit implementation overview

Theme	Subtheme	Example quotes
Awareness and reach	Familiarity amongst IPC frontline staff	**•** The CPE toolkit came out in early 2014. (…) I think it was very well publicised (IPC frontline staff 14). **•** I remember when I was a nurse on the shop floor I would have no idea and I only know because I’m in this job. (IPC frontline staff 15)
	Unfamiliarity amongst general frontline staff	**•** I’m not aware of anything other than what we’re doing at the moment. (General frontline staff 11) **•** I have heard of it but I haven’t read it. (General frontline staff 3)
	Awareness raising effect of outbreaks	**•** When we had our outbreak on the ward, so that’s how we became aware of it, from the infection control team. (General frontline staff 4)
	Multiple CPE toolkit communication channels	**•** Because I’m an infection prevention matron and it came through my manager. (IPC frontline staff 6) **•** Because somebody emailed it to me to read when we first came across CPE. (IPC frontline staff 3)
CPE toolkit implementation and impact pathway	Proximity to CPE as trigger for change	**•** It’s just an experience I don’t want to go through again but it’s been definitely an experience that obviously was needed to go through to learn from it. And we certainly have. And even though it’s been negative for patients to contract CPE, we’ve come out with a positive outcome that people have learnt and hopefully it won’t happen again. (General frontline staff 4)
	CPE toolkit as reference document	**•** All of our problems preceded the national guidance. (…) But obviously we’ve made sure that we were at least as a minimum in line with national guidance. (IPC frontline staff 1)
	Alignment with the CPE toolkit considering circumstances/Local adaptions	**•** The policy has been adapted as we’ve experienced more patients or the particular issues their nursing usually brings with them. (IPC frontline staff 21) **•** We’ve slightly moved the goalposts because of incidences we know that have happened. Incidents elsewhere, so those become alert areas.” (General frontline staff 20) **•** We used the guidelines what the suggestions were and if we thought it wasn’t enough then we tightened that suggestion up and put it into the policy. (IPC frontline staff 16) **•** Our policy is different in that in the toolkit it talks about isolation in the side rooms. Because we’ve had the problems that we’ve had we have an isolation ward. (IPC frontline staff 8) **•** We put a lot of emphasis on infection prevention, because it’s a cancer hospital and infection is something that kills cancer patients. They’re compromised and in the latter stages of disease. So we put a lot of emphasis on infection prevention, full stop. (IPC frontline staff 4)
	Interdisciplinary development process for local CPE plans	**•** We extracted what was relevant out of the CPE toolkit. There was a few of us that were looking at it, a couple of nurses and a microbiologist at the time. We used the toolkit quite heavily. (IPC frontline staff 15) **•** Well, obviously, any policy that we have would be a collaboration between [Pause]. Well, looking at national guidelines and discussing it within the team. We have a nurse consultant and myself who would pull it together and take it forward from there, and then we have a structure whereby we’d ratify it through the infection control committee. (IPC frontline staff 1)
	Multiple local CPE plan communication channels	**•** It was mentioned at meetings like [the] infection control committee, which has attendance from all divisions. There was email communication from top down so Chief Exec and Medical so that went to at least all the consultant bodies. I did talk with every divisional audit meeting. Infection Control committees. So that’s where it must have gone down to division. (IPC frontline staff 17) **•** We’ve spent a lot of time promoting it and talking about it. We’ve done loads of education on the ward. We’ve gone to the staff and we’ve invaded their handovers to speak about it and told them what to do, why it’s important. (IPC frontline staff 13) **•** We’ve done a lot of work in terms of publicising CPE around the Trust. So we did a video for example, just giving a short I think it’s a four minutes lecture on CPE. That’s hopefully casting. We showed that to all infection control practitioner training sessions. We produced some information sheets. (IPC frontline staff 2)
	Implementation challenge	**•** They [general frontline staff] know what they’re doing so they’re dealing with CPE, they’re managing it every day, they probably wouldn’t be able to tell you what it was. (General frontline staff 11) **•** It’s easy to write a policy you know, I’ve done that. It was easy to write the CPE policy, but actually how to implement it was a nightmare. (IPC frontline staff 19) **•** So, enforcing those, enforcing that change, making people aware of why we’re doing that is a… is still a challenge. (IPC frontline staff 5)
Views and responses to the CPE toolkit	Usefulness	**•** I think looking through them it was very useful, yeah. (IPC frontline staff 7) **•** I think the tool kit is actually quite comprehensive. There is always room for improvement, but actually it contains quite a lot of information and as I say the flow chart I think was very good and you have updated it I know a number of times and we have updated our policy in the light of that. (IPC frontline staff 9)
	Non-applicability	**•** There’s a lot of very fundamental practicalities. In theory, you can think, ‘Oh, this is fine. We have a side room; we can do this,’ and then you think, the shower is seven doors down – does that increase or reduce the contamination risks, insisting on showering? (IPC frontline staff 21) **•** I think it’s a guidance written around policy, written around assuming that everything takes place but it doesn’t seem to fit real life, the actual care that goes on. (IPC frontline staff 5)
	Lack of specificity and evidence base	**•** I think at the time they were quite useful but the more we had to sort of deal with it they were a little bit too vague. (…) I suppose if they were to put the guidelines out there and it was just so new and they couldn’t be specific because we were all learning about it weren’t we. (IPC frontline staff 16) **•** I think it’s the epidemiology that’s missing. (…) It does make you question the evidence that is informing those policy decisions, and it does for me. When I’m giving the education, it feels like a little bit hollow. And if anybody asks me a question I couldn’t answer it. (IPC frontline staff 6)
	Length and lack of accessibility	**•** I think initially when I first saw them I was terrified because they were really long and all the different appendices I found it really difficult to put together. If I was working on the ward I would not have been able to do it. (IPC frontline staff 19)
	Uncertainty about CPE toolkit target group	**•** There’s lots of information and people don’t read it, people won’t read it. You’ve got to make it more…simpler. I as an infection control nurse could understand that but I’m not completely sure that A, people have the time to read that on the wards and B, they completely understand what it is. So, it depends on who you’re directing it at. (IPC frontline staff 14)
CPE toolkit improvement suggestions	Updates including further evidence	**•** For the toolkit itself the time’s right for, following this evaluation, obviously, to re-address it. And to address how it fits into the UK – well, English – epidemiology now. Because I think this is a moving target, really, and we need to adapt. The guidelines are four years old already and the numbers have increased dramatically. Things have changed particularly, so I think that now’s the opportunity, once we have a feel for what the issues are from the toolkit itself, so it’s prime time to do this work. (IPC frontline staff 1) **•** I think it would be useful having a CPO document and highlighting the differences between CPO and CPE would be good. (IPC frontline staff 23) **•** It said here, the working group recognises when compared to other organisms (…) these were all being considered elsewhere. Now I think for someone like me I need to know where the elsewhere is. (IPC frontline staff 23) **•** It would be good to have sort of links to subject matter experts to be able to ask them sort of questions and interpret what we should do in certain situations. (IPC frontline staff 5) **•** I’d prefer a live update, a live updated website that I can refer my staff to so that they’re aware of where there are concerns and problems. (IPC frontline staff 5)
	Target group representative involvement	**•** The early iteration of the toolkit was not practical. The later one, the one that eventually came out, I’d like to think that’s because people like me who attended those meetings help shape the thing into reality. It became more useful and we used it as a basis for writing our policies. (IPC frontline staff 4)
	Emphasis on local adaptions and tailored information	**•** We will take the guidelines under advice and adapt them as appropriate for our local situation. I think some smaller trusts might feel it was more difficult to go against perceived national guidance. (…) I think you need to adapt it to what’s going on locally and use that as your basis and then justify how you’ve changed from it. If that was spelt out to people, and how they might change it if their circumstances were different, that might help colleagues out there who are maybe less sure of what to do. (IPC frontline staff 1) **•** If I had a little bit of abbreviated information with the most important aspects as regards the emergency department, then I can disseminate those to our doctors so they are more in the know. (General frontline staff 3)
	Additional visualisations	**•** I really like a world map, you know where you have got the different color-coded countries, where they have had problems etc., I think that’s really informative, I really like that and it gives a very visual picture to people as to the spread of this organism as to how rapidly its spreading in a very short time. (IPC frontline staff 9)

### Awareness and reach of the CPE toolkit

The extent to which frontline staff came into direct contact with the published CPE toolkit varied by staff group. All interviewed IPC frontline staff were aware of the CPE toolkit. This was rarely the case amongst general frontline staff (10%) and the few who recalled seeing it, acknowledged that they had not read it. General frontline staff, which referred directly to the CPE toolkit, usually did so in the context of CPE outbreaks. IPC frontline staff often could not remember how they initially heard about the CPE toolkit. If they did, this was typically through their professional networks and PHE mailing lists.

### CPE toolkit implementation and impact pathway

**Fig. 1:**
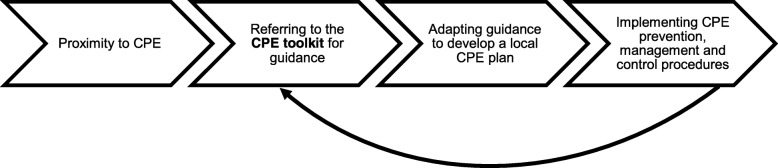
Typical CPE toolkit impact pathway

All trusts had local CPE plans in place and participants described CPE prevention, management and control procedures that aligned with the CPE toolkit guidance as much as possible considering local circumstances. Figure 1 provides an illustration of the role of the CPE toolkit in the typical development and implementation process of local CPE plans.

The process started with encountering CPE, either within their trust or becoming aware that it was a problem at other, particularly neighbouring trusts. CPE proximity seemed crucial in influencing the implementation process by raising awareness of CPE as a problem (82%). In response, IPC frontline staff would use the CPE toolkit to inform trust practice and translate it into local procedures and policies, making amendments to accommodate their circumstances and demands. Examples of local adaptations were enhancement of CPE precautions for high CPE colonisation rates or CPE outbreaks and large-scale patient cohorting at CPE isolation wards in response to insufficient isolation facilities. The three trusts that had local plans in place before the CPE toolkit was published reported their own hospital CPE outbreaks as triggers for developing CPE policies in the absence of national guidance. When the CPE toolkit was published, they referred to it to check if their policies aligned with recommended procedures, but did not necessarily find them useful.

The process of developing local CPE plans was often described as an interdisciplinary effort. Considering multiple perspectives helped to engage different staff groups and aimed to generate policies that were relevant, workable and acceptable for users. Communication channels for local CPE plans varied, including word of mouth, meetings and training sessions, intranet communications and written information leaflets and checklists. There was awareness that the implementation of procedures recommended in the CPE toolkit or locally-relevant adaptations promoted a general confidence in CPE prevention, management and control capabilities. This, however, did not necessarily co-exist with an in-depth knowledge of CPE, even in hospitals that experienced outbreaks.

The described process (Fig. 1) was iterative in nature without an ultimate endpoint, as responding to implementation experiences and situational factors (e.g. CPE outbreaks, availability of new evidence) local plans and procedures continued to change. Overall, developing local CPE plans was seen as less of a challenge than their implementation.

### Implementation facilitators and barriers

Table 4 provides an overview of factors mentioned by participants that acted as facilitators and/or barriers to implementing recommended CPE prevention, management and control procedures. They are grouped under the most relevant COM-B model component and illustrated by verbatim quotations. Factors influencing implementation often interacted as staff capabilities (e.g. knowledge and skills) and opportunities (e.g. social influences and available resources) had an impact on their motivation (e.g. beliefs and intentions) to engage in target behaviours.

**Table 4:** Factors influencing the implementation of recommended procedures

COM-B component	Theme	Facilitator/barrier	Example quotes
Capability: Psychological capabilities	Awareness and knowledge of CPE and relevant procedures	Facilitator/barrier	**•** I feel like I went from never hearing of it to maybe now having a basic knowledge of what we do and things and tests and things like that. (General frontline staff 19) **•** The toolkit is raising awareness at ward level so that people should be making this assessment for – well suspected cases of where there’s been a hospital admission in the last twelve months. (IPC frontline staff 20)
	Competing demands	Barrier	**•** CPE is competing against all these other national agendas. (General frontline staff 14) **•** From an infection control point of view, we’ve got to keep services running and we’ve got to balance the need to manage people safely but also be able to keep services going so that they get the treatment they need in a timely fashion. (IPC frontline staff 4)
	Uncertainty about CPE risk factors/effectiveness of screening	Barrier	**•** I think the other thing that comes up that can be slightly confusing is about which people have been in which hospitals, it’s quite loose isn’t it. (IPC frontline staff 12) **•** Well I mean they are, you know really motivated and quite dynamic in many areas but I think they have looked at other trusts like the [name of neighbouring trust] for instance and maybe they don’t think you pick enough up to warrant it so they’re doing a different risk assessment. (IPC frontline staff 11)
	Information overload	Barrier	**•** Sometimes these things are sent and you look at them and you think very good, there is an attachment and you don’t read it and you move onto the next email and off you go. It’s like most emails that I get, I don’t get time to read them. (…) We get an awful lot of documents and you have to be selective in what you think is the most relevant to your area or the most appropriate. (General frontline staff 3) **•** There was lots of it [communication]. There was lots of it and infection control did kind of a lot of roadshows and lots of sessions to talk about CPE. I’m not sure the general staff on the ward, if you were to ask them, would still be able to tell you what CPE was. (General frontline staff 11)
	Embedding CPE procedures into routine practice	Facilitator	**•** And also I think sometimes easier to make it a part of their job, so on and so forth possibly. (General frontline staff 1) **•** It’s embedded yeah but the other wards - the low risk wards - they know about it, they know what it means and if they’ve got a question or they need to call us then they get hold of us and we talk to them about it. With the high risk wards they’re so… It’s just engrained in their practice now. (IPC frontline staff 13)
Opportunity: Social influences	Multidisciplinary, trust-wide and across-trusts efforts and knowledge sharing	Facilitator/barrier	**•** If the patient’s been transferred through one of the normal wards we would make sure to have proper communication, proper handover to the team, make sure that we phone them. If they need to be isolated, that they need a side room and make sure that the nursing staff there will know and again, communicate to the whole team, to the doctors, to the porters and to the cleaners, to the nurses, to the HCs [home carers] so everyone knows what they are doing. So as a hospital we are working together to try to prevent it spreading. (General frontline staff 7) **•** If they are going into another care provider or district nursing or there is a lot of concern over looking after a patient who is CPE-positive in the community, and what that means to how those staff respond and react to that particular patient. (General frontline staff 20)
	Dedicated IPC staff	Facilitator	**•** Infection control on here, they come every morning so they guide us as to when we need new samples. (General frontline staff 21) **•** We contact the ward. We sort out the cleaning, the isolation, when they are screened, so we actually advise them step by step what to do and I think they do follow it. (IPC frontline staff 6)
	Supervision and leadership	Facilitator	**•** I think it’s partly about leadership in that if you demonstrate to your colleagues that you are adhering to this Trust policy, Trust procedure, as a senior doctor, they will then do the same. I can’t tell people to roll their sleeves up if my sleeves are down. (General frontline staff 3) **•** So yes, if medics get involved in implementing a policy, then it works more, it becomes more effective as well as nurses and healthcare assistants. When it is coming from the top, I feel it becomes more effective and people listen more. (General frontline staff 1)
	Management support	Facilitator	**•** Actually, the one thing I will say is they’ve [trust management] been incredibly supportive. They required costings but not a formal business case. It was more a case of, ‘Tell us how much this is going to cost so we can budget for it,’ as opposed to, ‘You submit a business case and we’ll see if we approve it or not.’ (IPC frontline staff 1) **•** I think what the toolkit did for us is it helped to focus the board, the board of directors on the importance of CPE and how we manage it and as a result of that the trust made a big investment in the CPE plan, programme. (IPC frontline staff 8)
	Social comparison	Facilitator	**•** We’ve heard of some Trusts as becoming so familiar with the CPE they’ve got as to almost become blasé, as to say, ‘Well, we’ve got it. We’re not gonna get rid of it.’ We haven’t reached that point. It’s still something we control; it’s still something we are aiming to isolate and prevent transfer. (General frontline staff 17) **•** From trusts in the region which obviously I can’t name I’m not sure whether they’re taking it as seriously as what we do. (IPC frontline staff 19)
	Patients’ lack of awareness and collaboration	Barrier	**•** I think it is that privacy and dignity of where you’re putting the swab, and people weren’t comfortable with that to start with, so that took a little bit of work. (General frontline staff 20) **•** We just have to explain it to them. I have encountered one who is just refusing to get it done because it’s per rectal. (General frontline staff 8)
Opportunity: Physical environment	Encountering CPE	Facilitator/barrier	**•** Obviously, infection control generally has a reputation for, one, potentially being a bit overzealous, I think, about something that clinicians don’t always see as a problem, but once they saw the nature of the resistance and the potential issues that we could have, I think it got them on-board fairly quickly. (IPC frontline staff 1) **•** If CPO was becoming a problem, I can imagine everyone knowing about it much more. (…) It would take a problem to get a robust procedure in place unfortunately. (…) I think it has to be recognised that we’re in the situation with the number of cases that we have that we’re certainly beyond the UK national guidelines by a long way. It’s not designed... It’s not fit for purpose, really, for us. (IPC frontline staff 23)
	Lack of side rooms	Barrier	**•** One thing that we found in our team quite difficult was the fact that the guidance was to isolate all suspected cases and I don’t think any acute hospital in England or the UK has enough side rooms to properly do that so I find sometimes we’re having to risk assess and then act upon that risk assessment. (IPC frontline staff 10) **•** What I thought of the toolkit? I mean I thought that we’d have a problem because there would be a lot of patients answering yes to the risk assessment and we are short of side rooms at the best of times. (…) So I did have concerns whether we could actually deliver that and implement it within the Trust. (IPC frontline staff 17)
	Availability of resources and financial investments	Facilitator	**•** I think we’ve got the resources we need. We do our audits. We do our meetings and we do our training. (General frontline staff 7) **•** Having an in-house laboratory I think is key, otherwise I think we would have not been so on top of the game. (IPC frontline staff 12)
	Age, layout and location of hospital	Facilitator/barrier	**•** I think in the new building it’s easier to isolate patients because all the side rooms are ensuite. The hospital that I cover is a very old building and not all the side rooms are ensuite. (IPC frontline staff 19) **•** So you can pop that up and get to all the data cables, all the water pipes and the services. Other trusts will have sealed ceilings. (IPC frontline staff 5)
	Staff shortage and high staff turn-over	Barrier	**•** We have a high turnover of staff particularly amongst doctors you know they change every six months. They might not always come across CPE in other trusts so we have a lot of education, do a lot of education with them. (IPC frontline staff 8) **•** We’re an area that often or frequently experience shortages in staffing. Therefore, it kind of leaves us heavily dependent upon agency and bank staff who maybe aren’t as robust, shall we say, in their procedures. (General frontline staff 11)
	Time pressure	Barrier	**•** Because people are busy so people don’t have time for teaching sessions. So yeah, I think that’s it really just ad-hoc teaching on wards so you capture the nurses. (IPC frontline staff 7) **•** I think it is probably because the medical staff is too stretched at the moment, and you know any additional responsibility given to them, I think you know makes it more difficult. (General frontline staff 1)
	CPE as part of wider IPC and AMR plan	Facilitator/barrier	**•** I think initially we went straight with a CRO, CPO protocol and actually it needs to fit more with gram negatives and then with a bit on CPO so it’s more embedded and less alien and less scary. (IPC frontline staff 19) **•** Before the CPE toolkit came out we had a general MDRGMB policy and I think the first iteration… let me get this right… I think we tried to incorporate the CPE guidance into that and it did two things really, it made the policy rather long which I think some people found inconvenient but certainly the feedback that we got from nurses, including the infection control nurses, was that it was too complicated and they wanted a separate CPE policy. (IPC frontline staff 22)
Motivation: Reflective motivation	Optimism, beliefs about CPE consequences and confidence in capabilities to manage it	Facilitator	**•** I am confident in my colleagues and I am confident the way the nurses observe their infection control management I think is very commendable, very good, they take on board what they are trained to do. I think the medical staff do a much better job than they used to. There is always some room for improvement, but there is definitely, there is definitely an improvement. (General frontline staff 3) **•** I do find it interesting because it’s just the fact that we are kind of leaning towards a post antibiotic era is quite scary prospect you know and how we can and what we can do and how we can do as part of the frontline I suppose to stop that and reduce that while we can. We’re not going to get completely rid of it but to reduce it down to you know manageable levels. (IPC frontline staff 14)
	Prioritising CPE	Facilitator	**•** It’s on the naughtiest list. (…) We manage them higher precautions than MRSA, the CPE, yeah. (General frontline staff 12) **•** We’ve got to make it clear to them that the CPEs are not the same as MRSA or something like that you know, they’re a different level of risk altogether. But at the same time I don’t want them to go away from that meeting or training session thinking, ‘Oh it’s alright we can just slip in MRSA patients anywhere because they’re not the CPEs. (…) I would certainly intend to have a zero tolerance to CPEs so we will do whatever we need to do to contain them and we’ll consider any intra-hospital transmissions unacceptable. (IPC frontline staff 22)
	Continuous improvement efforts and lessons learnt	Facilitator	**•** We developed, or relooked at our action plan for the management of CPE and by June we had extended our screening programme. July, August we opened up CPE isolation wards because we weren’t able to cope with the amount of patients. (IPC frontline staff 8) **•** I think it’s good to have those channels of communication and to feedback when things have gone well if you’ve had a complicated patient in and feeding that back, maybe doing an after action review, learning from what’s happened, what we can do better because I really think, one of the things we bring back in these after action reviews is that this is the future. (IPC frontline staff 10)
	Doubts about evidence base and practicality of recommended procedures	Barrier	**•** So we have moved away from the Public Health guidance document, because it hasn’t been updated for quite a while, so we’ve taken it that we will screen everybody from abroad. (General frontline staff 20) **•** I don’t know whether the toolkit’s really helpful if we actually have a problem in terms of the cluster or an outbreak. (IPC frontline staff 17)
	CPE prevention, management and control part of social professional role and identity, specifically to protect patients	Facilitator	**•** Being the deputy in charge most of the time I’m in charge of the unit so I do need to be aware of it. It’s part of my role to be aware that you can at any time get patients with CPO. (General frontline staff 7) **•** I’m the lead for the infection control team so responsibility to make sure that we educate staff and that we make sure they’re doing the right things in terms of CPE. (IPC frontline staff 4)
	Taking initiative and ownership of CPE prevention, management and control efforts	Facilitator	**•** Basically, we took ownership of our own problem and tried to deal with it rather than just relying on rules and regulations that were around the rest of the hospital. (General frontline staff 16) **•** We actively screen for it, and we’ve made a decision that we are going to do that. We’re not going to sit back or wait and see. (IPC frontline staff 21)
Motivation: Automatic Motivation	Concerns about CPE, its consequences and management	Facilitator/barrier	**•** I spoke to a colleague who happened to come here on a CPE accreditation inspection of the lab and they said that they were in a Trust where they had no idea how the CPE got out of control. That was really worrying to hear. He said that it just crept up on them and they couldn’t reduce the numbers despite multiple outbreak meetings and so on, so we are very proactive when we have multi-resistant organisms. (IPC frontline staff 23) **•** In one way there’s not the fear, so that’s quite good, you know, people aren’t scared, if they get it they aren’t scared of it. But maybe if there was that awareness raising about CPE then it might encourage better compliance with hand hygiene or, you know, things that we can do to prevent it. (General frontline staff 11)

Due to the novelty of the topic, a lack of awareness and knowledge especially amongst general frontline staff prevailed. Expert CPE knowledge went hand-in-hand with concerns about resulting risks and increased efforts to deal with CPE. Uncertainties about CPE risk factors and its management as well as receiving too much and too detailed information were problematic. Implementation was made easier, if new processes such as screening procedures were added to already existing standard operational procedures and routines. One way of doing this was making CPE part of a wider IPC plan. This however posed the risk of not paying enough attention to CPE but prioritising other more pressing issues.

Contextual factors, including resources and social influences, were overall key to implementation. Participants recognised multidisciplinary, trust-wide and across-trusts efforts and knowledge sharing as facilitators, even if these slowed down the process since involving various stakeholders took time. This approach promoted communication of relevant patient information and helped to creatively deal with important barriers, such as insufficient numbers of isolation facilities. A problem mentioned was communication and collaboration with community care providers, where anxieties and a lack of CPE knowledge hampered the discharge of colonised patients.

Further facilitative social opportunity factors included the presence of dedicated IPC staff and support from supervisors, modelling good practice, and trust management support. The CPE toolkit was seen as a credible source of information, which could help to secure management support for financial investments to address resource challenges such as a shortage of isolation facilities or funding for additional training. Comparing their own situation to other trusts seemed to encourage CPE prevention and control efforts as CPE outbreaks at other trusts highlighted the seriousness of the problem; some participants mentioned a desire to outperform other trusts. Patients’ lack of awareness and refusal to participate in recommended procedures, particularly refusing rectal swabs, was a barrier reported by some trusts but could be addressed with communication and reassurance.

Encountering CPE increased staff motivation to develop local plans and follow recommended procedures and led to raised awareness and CPE knowledge. On the other hand, if CPE was already a problem, procedures outlined in the CPE toolkit were often not seen as adequate or practicable, forcing staff to find new ways of managing the problem, including cohorting whole patient groups. Older hospital buildings were a barrier to ensuring appropriate cleaning of facilities and isolation. Staff shortages, time pressures and competing IPC demands complicated matters further. Training members of staff for example, the most frequently named dissemination strategy for local CPE plans, was difficult due to high staff turnover and work pressures. There was increased anxiety in the context of resource shortages as this increased the challenge of managing potential CPE cases. Being extremely concerned and anxious about CPE seemed to evoke helplessness and inaction.

Optimism and confidence in the capability to carry out recommended procedures was both a barrier and facilitator. A certain level was required for successful implementation, for example when carrying out rectal swabs. An over-confidence, assuming current practice based on clinical expertise and experience sufficed, however, prevented staff from acquiring further knowledge and skills. Some staff mentioned that the CPE toolkit helped reduce concerns and improved beliefs in their capacity to manage outbreaks as it provided clear guidance. Continuous improvement efforts, e.g. using monitoring, audit or feedback, helped adapting patient management procedures. IPC nurses typically took charge as they felt responsible for and took ownership of the issue and for passing on relevant knowledge. Overall, it was facilitative to change if staff perceived following recommended procedures and dealing with CPE as an important part of their role and identity.

### Views and responses to the CPE toolkit

Participants, who were familiar with the CPE toolkit, often could not remember their initial thoughts or recall its specific content, but reported views were mixed (Table 3). Some thought it was comprehensive and useful and much needed at the time, especially the flowchart, while others perceived the recommended procedures as impractical to implement locally. Participants criticised the CPE toolkit for not being specific enough, sometimes acknowledging this could be due to lack of a clear evidence base. The lack of scientific evidence led others to question the credibility of the information provided and to voice distrust in or confusion around the recommended procedures. While the toolkit did not include enough information for some, other participants criticised it for being too long, making it inaccessible. There was also some uncertainty about the CPE toolkit target group.

### CPE toolkit improvement suggestions

The most commonly voiced improvement suggestion was that the current version of the CPE toolkit should be updated regularly (23%). Participants were keen that an updated version would include new evidence and further clarifications on the difference between CPE and Carbapenemase-producing Organism (CPO), risk factors and screening procedures, including different implementation options to fit clinical practice. One way to address concerns around the evidence base of the CPE toolkit and strengthen its credibility could be linking it to related guidance and research publications or providing contact details of experts for further queries. A better fit for clinical practice might be achieved building on previous involvement of experts “on the ground”.

Participants felt it needed to be more explicitly highlighted that the CPE toolkit encouraged local adaptations and to provide tailored information to meet the needs of different staff groups and types of trusts, for example trusts with low and high colonisation rates. To improve accessibility and reduce information overload for general frontline staff, an abbreviated version was suggested. Participants also expressed a desire for further visualisations, particularly in the form of graphs and figures concerning epidemiological information. Those figures would not necessarily need to be provided in writing, but could be accessible online and should again be updated regularly. Table 5 summarises recommendations for improving the CPE toolkit based on participants’ suggestions and outlined implementation facilitators and barriers.

**Table 5:** Recommendation summary for future CPE toolkit versions

Recommendations	
• Regular revisions and updates with trust representative engagement	
• Additional clarifications specifically concerning CPE risk factors and differences to other infections	
• References to related guidance and additional scientific information sources	
• Visual information presentation, for example epidemiological maps and summary pages	
• Reassessment of the CPE toolkit target audience, considering different staff groups and their needs	
• Tailored information components clearly signposted to relevant audiences	
• Implementation guidance	

## Discussion

In this qualitative implementation evaluation, frontline staff representing a variety of roles and different hospital settings, in terms of CPE colonisation rates, time point of introducing local CPE plans, trust size and geographical region, were interviewed to examine in-depth their experiences concerning awareness and uptake of the CPE toolkit and recommended procedures. A minority of frontline staff had seen the published CPE toolkit and of those who had almost all were staff with specific IPC responsibilities. The majority of frontline staff was exposed to the information in the CPE toolkit indirectly via local CPE policies, which were in place at all participating trusts.

Interviewed IPC frontline staff typically used the toolkit as a resource or were aware that it had been used to inform the development of their local CPE plan. In cases where outbreaks preceded the CPE toolkit, it was used to check whether existing plans aligned to recommended procedures. The previously conducted survey with executive-level staff found that out of 99 represented trusts 64% said they had used the CPE toolkit to inform their local CPE plan whilst 32% reported having used it as provided [19].

Proximity to CPE in terms of local experiences or outbreaks at trusts nearby seemed to be a crucial facilitator of change via increased awareness and motivation. Specifically trusts with high colonisation rates and outbreak experiences were quick to have management and control procedures in place, even before the publication of the CPE toolkit that was not necessarily seen as useful anymore. This is in line with findings from the executive staff survey and research on regional CPE prevalence at English hospitals [19, 45]. The development of local plans and the introduction of CPE prevention, management and control procedures based on the CPE toolkit in response to such trigger events represent a multilevel implementation process typical for healthcare interventions in complex settings [46, 47]. It was not a strictly linear, rational and clock-workstyle pathway to change standard operational procedures. As found in research on introducing new procedures and systems in primary care [48], pathways to implementation were complex and both organisational and interpersonal factors, such as senior management support, a culture of knowledge sharing and multidisciplinary collaborations, mediated the process.

The evaluation captured the multidisciplinary nature of both, local plan development and their implementation. Continual efforts were required to adapt procedures in the light of changing conditions, such as increased experiences of CPE. The conceptualisation of the CPE guidelines as a toolkit allowed for differences between trusts and shifted the implementation focus from fidelity to local adaptations, which could be tailored to the specific contexts and built on local expertise. Such multicomponent approaches that build on and increase existing infection control measures have been found to be particularly effective in controlling CPE outbreaks, whilst feedback loops involving various stakeholders within the organisation are important to translate guidelines into implementable procedures [3, 46, 47].

Overall, writing local plans was not a problem, but difficulties arose implementing them. This is not unusual as even evidence-based guidelines are frequently not implemented in practice [49, 50] and therefore implementation research is crucial to understanding the reasons for this to improve uptake [46]. This implementation evaluation highlighted implementation barriers and facilitators that should be considered in future revision of the CPE toolkit. Furthermore, as epidemiological studies assessed CPE as a representative example for new emerging AMR threats [45], current findings could be helpful when developing and implementing other AMR focused policies.

Social and resource factors were the most important implementation facilitators and barriers. Organisational cultures were found to be particularly important for the implementation of new policies in complex organisations such as hospitals where many different groups of people interact [51]. Interview participants commented, for example, on the facilitative influence of senior management support, which is in line with the prior executive staff survey [19]. Acute trusts that placed a low priority on CPE prevention, management and control also had poorer compliance at the frontline.

Organisational factors are even thought to outweigh the impact of the quality of the evidence supporting new policies or procedures in respect to implementation [52, 53]. This may be partly because frontline staff did not necessarily have contact with the actual guidance, but rather with the local interpretation of them. Staff commented on the benefit of knowledge sharing and multidisciplinary efforts, similar to evidence on successful CPE outbreak control measures, which included dedicated IPC frontline staff and hospital-wide contact tracing as part of a multifaceted intervention [54]. Delegating overseeing the implementation of local CPE prevention, management and control procedures to specific IPC frontline staff seemed easier in large trusts with extensive IPC teams, while multi-disciplinary collaborations were facilitated in small trust by staff being familiar with each other and existing work relationships. Cross-trust coordination of IPC activities within regions, e.g. by sharing information about patients’ colonisation status between health-care institutions, seems particularly important in England and other countries where hospitals are connected by patient movements and inter-hospital spread is likely, and where there is the possibility of community associated CPE [26, 45, 55, 56]. A big resource-related challenge was staff having to negotiate competing demands with little scope to be released from day-to-day duties for example for relevant training. Keeping up with training was an even bigger issue if there was high staff turnover and employment of agency staff.

In the absence of cases, CPE concerns amongst participants were often low and so was the perceived risk to patients’ safety, hindering compliance with recommended procedures. This is supported by research that has found a poor compliance with guidance at the frontline if staff deemed their patients not at risk [57] and if there was a lack of fit between clinicians’ experiences and recommended practices [58]. Some participants at trusts with currently no or low numbers of CPE cases felt confident in their abilities to manage small numbers of cases but were less optimistic if they expected an increase in the future. This mirrors the perception of executive trust staff, who also voiced a lack of confidence in the value of the CPE toolkit when dealing with outbreaks [19]. Interview participants had general concerns about the effectiveness of the CPE toolkit guidance and questioned its evidence-base.

Although the CPE toolkit suggests a multifaceted approach in line with best practice evidence [3] and its overlap with other guidance based on expert consultations and systematic reviews [59, 60], evidence gaps remain. There is uncertainty for example with respect to CPE risk factors, cost effective surveillance strategies and the general robustness of the available evidence. It is important to address these gaps, although improving the evidence base alone will not be enough to strengthen guidance implementation [52, 53], which also depends on the described motivational and organisational factors.

Regular intervention revisions and updates are required to achieve sustainable change, but excessive guideline revision frequencies should be avoided as they may undermine implementation [46, 47, 58]. In the case of the CPE toolkit and in light of the influence of CPE proximity on implementation, it may be that maps providing epidemiological data on the geographical distribution of CPE positive isolates could be produced and updated frequently online, while the CPE toolkit in its entirety should only be updated when a considerable amount of new evidence is available [45, 58]. The need for comprehensive summary information should be considered when presenting new evidence and references and additionally information sources should be clearly signposted to accommodate further information needs. It would also be helpful to link the CPE toolkit to related guidance such as the CPE toolkit for non-acute and community settings [61], which would be important for issues around transfers to community settings.

The CPE toolkit contained information targeting different groups, including hospital frontline staff, board and executive-level staff and patients [10]. The evaluation found that it was used mainly by IPC specialist staff and disseminated to other frontline staff was via local plans and processes such as on the job training. It may therefore be useful to target the CPE toolkit for use by the IPCT, and provide them with recommendations and resources on how to disseminate its content to the hospital workforce. This approach, providing implementation guidance, would harness the responsibility felt by IPC staff to inform others. Additional staff groups such as microbiologists or pharmacists may also be involved in dissemination. A section for board level executives should remain due to the importance of garnering management support. The CPE toolkit could function as a comprehensive reference document, but with sections and resources clearly signposted to relevant audience (information for general frontline staff, microbiologists, pharmacists, management etc.) and with information specifically prepared for each target group to reduce information load and improve processing. A modular design could also consider different or incremental CPE incident levels as trusts with none or low colonisation rates require different action plans than those with widespread outbreaks. For low-prevalence CPE settings enhanced infection control measures even without active surveillance could suffice whereas hospital-wide contract tracing would be required to control outbreaks [54, 62].

Implementation guidance could be included in the CPE toolkit to facilitate the process of incorporating recommended procedures into clinical practice. A task force or implementation team consisting of different target group representatives and tools for conducting stakeholder analyses or leadership support assessments are promising options [63, 64]. Leadership support could further be encouraged, if necessary, by providing information on cost savings due to outbreak prevention measures [65, 66]. Overall, acute trust representatives should be involved in the revision of the guidelines and multidisciplinary collaborations at trust level should be encouraged to revise local policies. Involving end-users promotes implementable recommendations and a sense of ownership and commitment to adherence [58].

Drawing on behavioural science theories, this implementation evaluation contributed to addressing a lack of rigorous, theory-based implementation evaluations and provided an evidence base for updating the CPE toolkit [17, 50]. The in-depth, qualitative research approach allowed for flexibility to explore emerging issues and new points of views, which was particularly important to understand the implementation in context and to uncover not only the intended, but also unintended intervention consequences [46, 47]. Taking into account participants’ responses and following up on their specific experiences and implementation concerns meant that topics outlined by the interview guide were not assessed in a standardised, quantifiable but holistic manner [39, 67].

A personal access strategy and entering the field during data collection [27, 68] resulted in good participant engagement rates with 12 out of 14 trusts and general as well as IPC frontline staff participating. Purposively recruiting trusts and frontline staff using a maximum variation sampling approach [29–31] provided insight into wide range of frontline experiences in different settings. The recruitment was based on the previous survey sample [19] and required consent to interviews, possibly selecting trusts already being more engaged in the CPE toolkit implementation than non-survey participants. Considering late adopters, trusts that introduced local CPE plans only with a delay of up to 3 years, might counteract this potential selection bias to some degree. With telephone interviews being conducted over multiple days, answers concerning awareness of the CPE toolkit could have been affected due to trust internal information exchange, but remained overall low amongst general frontline staff.

The CPE toolkit was nationally disseminated and there were therefore no examples of acute trusts not provided with it to be considered for comparative purposes. Due to the multicomponent nature of the CPE toolkit, the evaluation focused on general, top-level implementation facilitators and barriers to provide generic improvement suggestions. Analysing the implementation of all target behaviours suggested by the CPE toolkit would have been beyond the scope of this research and would not have been reasonable considering the guidelines allowed for local adaptations. This should, however, be kept in mind when interpreting the findings and it would be useful to triangulate them with experimental research investigating the impact of the guidelines on specific target behaviours [69]. It would have been beneficial to conduct this implementation evaluation sooner after the national launch of the CPE toolkit, accompanying the implementation phase, to avoid potential memory biases. This approach would fit with a continual cycle of evaluation, feedback and implementation as subsequent adaptation is important for future guidance.

## Conclusions

The CPE toolkit was found to be helpful as a reference document for IPC frontline staff using it to inform and check their own CPE prevention, management and control plans in particular in the event of an outbreak. Frontline staff felt there was a need for an updated version of the CPE toolkit, including new evidence and guidance on how to tailor it to different contexts. Greater consideration should be given to implementation when developing national guidance and policy.

## Additional files

Additional file 1: Flowchart of the evaluation frameworks and research process linkage. (PDF 316 kb)

Additional file 2: Interview participation per trust. (PDF 67 kb)

Additional file 3: Interview Topic Guide. (PDF 268 kb)

## Data Availability

The datasets generated and analysed during the current study are not publicly available for data protection reasons but anonymised excerpts are available from the corresponding author on request.

## Abbreviations

AMR: Antimicrobial resistance
BCW: Behaviour Change Wheel
COM-B model: Capability, Opportunity, Motivation and Behaviour model
CPE: Carbapenemase-producing enterobacteriaceae
CPO: Carbapenemase-producing Organism
ECDC: European Centre for Disease Prevention and Control
IPC: Infection prevention and control
IPCT: Infection prevention and control teams
MRC: Medical Research Council
NHS: National Health Service
PHE: Public Health England
TDF: Theoretical Domains Framework
WHO: World Health Organization
