# Supplementary material for: Implementing a toolkit for the prevention, management and control of carbapenemase-producing Enterobacteriaceae in English acute hospitals trusts: a qualitative evaluation
Source: BMC Health Serv Res. 2019 Oct 12;19:689. doi: 10.1186/s12913-019-4492-4 (PMC6790044; doi:10.1186/s12913-019-4492-4)
Supplement: Supplementary file 1 — Flowchart of the evaluation frameworks and research process linkage. (PDF 316 kb) [file 12913_2019_4492_MOESM1_ESM.pdf]

## Supplement 1 – Flowchart of the evaluation frameworks and research process linkage

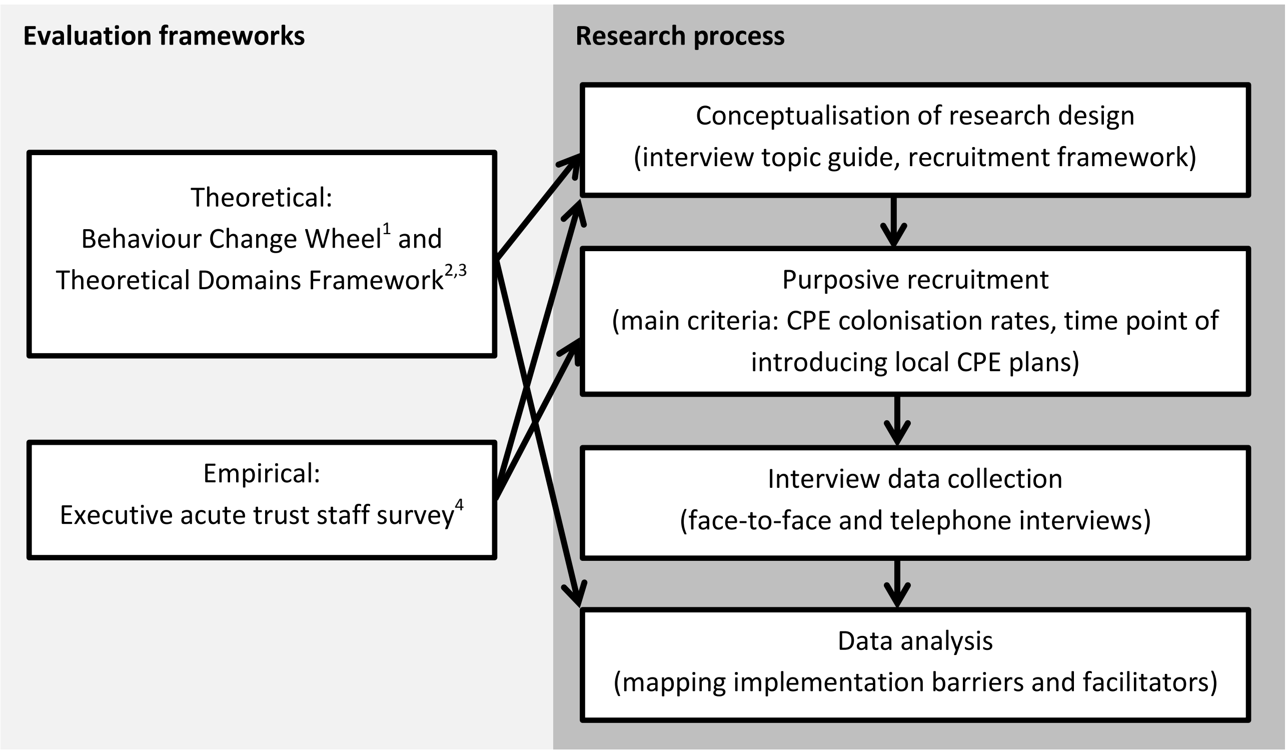


*Figure 1: Illustration of the evaluation frameworks and research process linkage.*

---

[1]Michie S, van Stralen MM, West R, Grimshaw J, Shirran L, Thomas R, et al. The behaviour change wheel: a new method for characterising and designing behaviour change interventions. Implement Sci. 2011;6:42.

[2]Atkins L, Francis J, Islam R, O'Connor D, Patey A, Ivers N, et al. A guide to using the Theoretical Domains Framework of behaviour change to investigate implementation problems. Implement Sci. 2017;12:77.

[3]Cane J, O'Connor D, Michie S. Validation of the theoretical domains framework for use in behaviour change and implementation research. Implement Sci. 2012;7:37.

[4]Coope CM, Verlander NQ, Schneider A, Hopkins S, Welfare W, Johnson AP, et al. An evaluation of a toolkit for the early detection, management, and control of carbapenemase-producing Enterobacteriaceae: a survey of acute hospital trusts in England. J Hosp Infect. 2018;99:381–9.
