# Supplementary material for: Implementing a toolkit for the prevention, management and control of carbapenemase-producing Enterobacteriaceae in English acute hospitals trusts: a qualitative evaluation
Source: BMC Health Serv Res. 2019 Oct 12;19:689. doi: 10.1186/s12913-019-4492-4 (PMC6790044; doi:10.1186/s12913-019-4492-4)
Supplement: Supplementary file 2 — Interview participation per trust. (PDF 67 kb) [file 12913_2019_4492_MOESM2_ESM.pdf]

Supplement 2 – Interview participation per trust

▪ Trust 1 (no CPE cases / pre-CPE toolkit):	2 interview participants
▪ Trust 2 (no CPE cases / early adopter):	4 interview participants
▪ Trust 3 (no CPE cases / late adopter):	5 interview participants
▪ Trust 4 (some CPE cases / pre-CPE toolkit):	5 interview participants
▪ Trust 5 (some CPE cases / early adopter):	5 interview participants
▪ Trust 6 (some CPE cases / early adopter):	2 interview participants
▪ Trust 7 (some cases / late adopter):	2 interview participants
▪ Trust 8 (many CPE cases / pre-CPE toolkit):	3 interview participants
▪ Trust 9 (many CPE cases / early adopter):	5 interview participants
▪ Trust 10 (many CPE cases / early adopter):	2 interview participants
▪ Trust 11 (many CPE cases / late adopter):	5 interview participants
▪ Trust 12 (many CPE cases / late adopter):	4 interview participants
