# Supplementary material for: Implementing a toolkit for the prevention, management and control of carbapenemase-producing Enterobacteriaceae in English acute hospitals trusts: a qualitative evaluation
Source: BMC Health Serv Res. 2019 Oct 12;19:689. doi: 10.1186/s12913-019-4492-4 (PMC6790044; doi:10.1186/s12913-019-4492-4)
Supplement: Supplementary file 3 — Interview Topic Guide. (PDF 268 kb) [file 12913_2019_4492_MOESM3_ESM.pdf]

Supplement 3 - Interview topic guide

Topic	Interview guide
**Introduction:**<br>**Purpose of research project, audio recording procedures and signing consent forms**	▪ The purpose of this study is to improve our understanding of how carbapenemase-producing Enterobacteriaceae (CPE), colonisations and infections, are being prevented and controlled within acute trusts in England.<br>▪ Before we get started, I'd like to tell you that I will be recording the conversation to get an accurate record of what we discuss. You can ask for the recording to be stopped at any time. What you say will be kept confidential and anonymous.<br>▪ Can I check that you have received and read a copy of the information for participants?<br>▪ Do you have any questions about it?<br>▪ I am going to start the audio recording now and begin the interview.
**Interviewee CPE/IPC role**	▪ Can you tell me if you have any specific responsibility around CPE or IPC in your current role, and briefly describe this?
**Significant changes to CPE prevention and control procedures**	▪ Are you aware of any significant changes to procedures for preventing and controlling CPE over the last 3 years at your trust, and if yes, describe them briefly?<br>→ **Prompts to be used if needed:**<br>- Do you know why these changes were made?<br>- Do you think these changes have, or will make a difference in the future?
**Influences on CPE prevention and control procedures**	▪ Are you aware of any procedures, guidelines or plans that are in place at your trust to<br>a) identify and prevent b) control and c) manage CPE?<br>▪ If aware, do these form part of procedures, guidelines or plans for MDRO or are they specific for CPE?
Physical opportunity: environmental context	▪ What resources are available to you for the purpose of preventing, managing and controlling CPE?<br>→ **Prompts to be used if needed:**<br>- E.g. training, materials, people?<br>- IPC team?<br>- Are isolation facilities available? Explore alternative means of isolation other than single rooms (patient and staff cohorting).<br>- Do you have enough capacity / time to screen patients?<br>- Has training on how to carry out the procedures in the plan been provided?<br>→ **Prompt for board level / managerial staff:**<br>- Has your trust invested in isolation facilities or provided financial support for relevant training?
Psychological capabilities: CPE knowledge and skills	▪ How would you describe your level of knowledge about CPE including how to manage CPE?<br>→ **Prompts to be used if needed:**<br>- If you had a CPE case or outbreak, what, or who, would help you to make decisions how to manage CPE?<br>▪ Do you believe you have the skills you need in order to manage CPE?<br>→ **Prompts to be used if needed:**<br>What would be required to improve your skills?

Supplement 3 - Interview topic guide

Reflective and automatic motivation: emotions, attitudes and beliefs about consequences and capabilities<br><br>*Social opportunity: Social influences*	▪ Are you personally concerned about CPE? And reasons for this?<br>→ **Prompts to be used if needed:**<br>- Are you concerned about a failure to manage it appropriately?<br>▪ Do you feel a) CPE is a problem at your trust b) confident in current procedures?<br>▪ Do you personally feel a) competent and b) confident to detect, manage and control CPE?<br><br>▪ Do you think CPE is a concern for other trusts in England?<br>▪ Do you think your colleagues are concerned about CPE and its management?<br>▪ Do you think your patients are concerned about CPE and its management?
**CPE toolkit awareness / reach**	▪ Have you heard of the guidelines called "Acute trust toolkit for the early detection, management and control of Carbapenemase-producing Enterobacteriaceae"?<br>▪ How did you become aware of the CPE toolkit guidelines?<br>→**Prompts to be used if needed:**<br>- Was there a launch event for the CPE toolkit?<br>- Did you receive any written communication from the trust and others, e.g. PHE (e.g. email, letters) about the CPE toolkit?<br>- Did you hear about the CPE toolkit by word of mouth (e.g. colleagues/manager/ etc.)?<br>- Did/do the senior members of staff / managers advocate the CPE toolkit?<br>→**Prompts for ey IPC informants only**<br>- Were you involved in developing your trusts guidelines for CPE?<br>- If yes, has the CPE toolkit been used in developing a local action plan to prevent, control and manage CPE at your trust?
**Responses to and interactions with the CPE toolkit**<br><br>**Facilitators and barriers**	***If participants are aware of the toolkit.***<br>▪ What do you personally think about the toolkit?<br>▪ How was the CPE toolkit and recommended procedures received at your trust?<br>→ **Prompts to be used if needed:**<br>- How did members of staff react towards the CPE toolkit when it was launched?<br>- Can you recall any patients' reactions to CPE toolkit guidelines admission procedure?<br><br>***If the person has used the CPE toolkit guidelines or aspects of them:***<br>▪ What has helped you to use the CPE toolkit?<br>→ **Prompts to be used if needed:**<br>- Easy to use / follow?<br>- Are any parts of it particularly useful?<br>- Did you receive any training?<br><br>***If CPE toolkit was not / only partially adopted:***<br>▪ Can you tell me why you and/or your colleagues have not adopted the toolkit / action plan developed by your trust as result of the toolkit?<br>▪ What barriers are you experiencing using the CPE toolkit?

Supplement 3 - Interview topic guide

	▪ What could be improved to increase the uptake of CPE management steps recommended by the CPE toolkit within your trust?<br>▪ Is there room for further improvement of the CPE toolkit to improve its usability?
**Fidelity / dose / adaptations**	▪ The CPE toolkit includes a flowchart specifying steps and procedures to control and manage CPE. Have you seen this flowchart and what do you think about the flowchart a) in general and b) each step?<br>→ **Prompts to be used if needed:**<br>- Have the steps outlined in the flowchart been tailored / adapted at your trust?<br>- Do you know how many patients are screened for CPE at your acute trust and how screening is implemented locally [i.e. ward or trust – whatever is relevant to the participant]?<br>- Does your trust use a risked based screening of all patients or focuses screening on specific high risk wards?<br>- Are the main recommendations of the CPE toolkit guidelines regarding screening and isolation followed?
**Close**	**End of audio recording**<br>▪ Thanks for participating.
